# Metamaterial Waveguide Devices for Integrated Optics

**DOI:** 10.3390/ma10091037

**Published:** 2017-09-05

**Authors:** Tomohiro Amemiya, Toru Kanazawa, Satoshi Yamasaki, Shigehisa Arai

**Affiliations:** 1 Institute of Innovative Research (IIR), Tokyo Institute of Technology, Tokyo 152-8552, Japan; arai@pe.titech.ac.jp; 2 Department of Electrical and Electronic Engineering, Tokyo Institute of Technology, Tokyo 152-8552, Japan; kanazawa.t.aa@m.titech.ac.jp (T.K.); yamasaki.s.ae@m.titech.ac.jp (S.Y.)

**Keywords:** metamaterials, integrated optics, III-V semiconductors

## Abstract

We show the feasibility of controlling the magnetic permeability of optical semiconductor devices on InP-based photonic integration platforms. We have achieved the permeability control of GaInAsP/InP semiconductor waveguides by combining the waveguide with a metamaterial consisting of gate-controlled split ring resonators. The split-ring resonators interact magnetically with light travelling in the waveguide and move the effective relative permeability of the waveguide away from 1 at optical frequencies. The variation in permeability can be controlled with the gate voltage. Using this variable-permeability waveguide, we have built an optical modulator consisting of a GaInAsP/InP Mach–Zehnder interferometer for use at an optical communication wavelength of 1.55 μm. The device changes the permeability of its waveguide arm with controlling gate voltage, thereby varying the refractive index of the arm to modulate the intensity of light. For the study of variable-permeability waveguide devices, we also propose a method of extracting separately the permittivity and permeability values of devices from the experimental data of light transmission. Adjusting the permeability of optical semiconductors to the needs of device designers will open the promising field of ‘permeability engineering’. Permeability engineering will facilitate the manipulation of light and the management of photons, thereby contributing to the development of novel devices with sophisticated functions for photonic integration.

## 1. Introduction

The photonic integrated circuit (PIC) is an on-chip photonic system consisting of various optoelectronic devices such as light sources, modulators, isolators, and detectors that are monolithically integrated and interconnected on a semiconductor substrate. It has made remarkable progress in the past few decades with developing applications and many contributions in the fields of optical communication, sensing, and imaging. At present, great attention is paid to scale-down, lower power consumption, and increase functions of optoelectronic devices to make next-generation PICs with high-density integration and high-level functionality. To develop such advanced photonic devices, we have proposed controlling the permeability of semiconductor materials used to make the devices [1,2].

The most important material parameters for photonic devices are the electric permittivity *ε* and the magnetic permeability *μ* of semiconductors used to make the device. Of the two, we can easily control and vary the permittivity to meet the needs for device operation because we can use various semiconductors with different permittivity values in a device. In contrast, the relative permeability of semiconductors is fixed to 1 at optical frequencies and difficult to move away from 1 because the magnetization of natural materials cannot follow the alternating magnetic field of light. In consequence, we can control only one parameter, permittivity, and this greatly restricts the flexibility in the design of photonic devices. If we can control the optical permeability, as well as the permittivity, of semiconductors, we will be able to develop new photonic devices far superior to existing ones.

The advent of metamaterials can remove the restriction on permeability. The metamaterial is an artificial material designed to have properties that are not found in nature. It is an assembly of periodically arranged multiple nanostructural elements, such as minute resonators, that interact with the electromagnetic field of light. The metamaterial can exhibit extraordinary permittivity and permeability values not existing in nature and can show even negative permittivity and permeability [3,4]. Such flexibility in designing *ε*-*μ* values enables to develop innovative technologies for sophisticated optical manipulation such as sub-diffraction-limit lenses, the storage of broadband light, and invisibility cloaking [5,6,7,8,9,10]. Metamaterials also have the potential to develop conventional optical devices into advanced, functional photonic ones to open a new field called metaphotonics [11,12,13,14]. To contribute toward the development of metaphotonics, we have proposed using metamaterial to break the permeability restriction. We have also confirmed that a semiconductor layer attached with a metamaterial can behave as a novel semiconductor, or ‘metasemiconductor,’ whose optical permeability can be controlled with an external electric signal. This is described in detail in the following sections.

The most simple and feasible application of controllable permeability is probably the efficient modulation of the refractive index of semiconductors. To vary the refractive index *n* in existing devices, only permittivity is modulated (*ε* → *ε* + Δ*ε*) with an external signal, using electro-optic effects. A change Δ*n* in the refractive index is therefore given by *n* + Δ*n* = (*ε* + Δ*ε*)^1/2^. Next, if we can modulate permeability (*μ* → *μ* + Δ*μ*) in addition to permittivity, we will be able to obtain a larger change Δ*n* given by *n* + Δ*n* = (*ε* + Δ*ε*)^1/2^(*μ* + Δ*μ*)^1/2^. This can be used to create highly-efficient and small-sized optical modulators for PICs. In the following sections, we describe the design, fabrication, and measurement of this permeability-controlled modulator. Controllable permeability has more potential applications for metaphotonics and next-generation PICs; wide-angle optical beam deflection using a semiconductor superprism is an example, which is mentioned in the Summary.

## 2. Metamaterial Waveguide Theory and Method of *ε-μ* Extraction

### 2.1. Split-Ring Metamaterial Compatible with Waveguide Optical Devices

In the field of photonic integrated circuits, the III-V compound PIC, more specifically the GaInAsP/InP-based PIC, is a leading part because it can provide the monolithic integration of various photonic devices including light sources [15,16,17]. Therefore, we now discuss a device structure that is compatible with GaInAsP/InP-based PICs. Our proposal can also be applied to silicon PICs.

We first decide the basic structure of metamaterial to control the permeability of photonic devices. Almost every photonic device used in PICs has the form of a planar semiconductor waveguide. Therefore, our purpose is to control the permeability of the semiconductor waveguide, using a metamaterial that is compatible with the waveguide structure. Most PICs are designed to operate with TE-polarized light, so the metamaterial has to interact with the magnetic field of TE light travelling in the waveguide.

**Figure 1:**
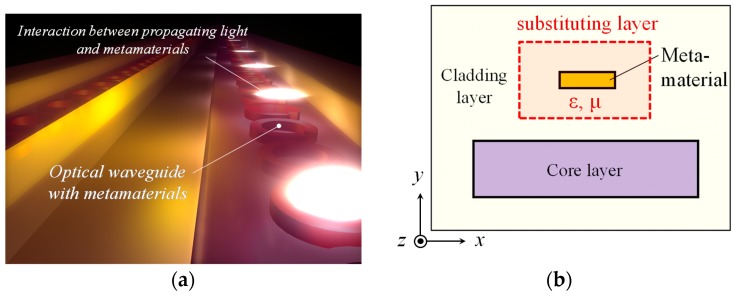
(**a**) Schematic image of optical waveguide with metamaterials on its surface. Magnetic interaction between metamaterials and light moves relative permeability of waveguide away from 1; (**b**) One example of cross section of the waveguide with the substituting layer.

The split-ring metamaterial is suitable for our purpose. It is an array of minute metal rings arranged periodically with a pitch smaller than the wavelength of light. An individual ring is an enclosed loop with one or more gaps and operates as an LC resonant circuit consisting of an inductor formed by the ring and a capacitor by the gaps [18]; therefore it is called a sprit-ring resonator, or SRR. The split-ring metamaterial consisting of a single-layer SRR array is quite compatible with planar waveguides because it can be easily formed on the surface of the waveguide (see Figure 1a).

In a semiconductor waveguide combined with the SRRs on its top surface, the magnetic field of TE light links with the SRRs. If the frequency of the light is nearly equal to the resonance frequency of the SRR, a circulating current is induced in the SRRs, and this produces a magnetic dipole moment in response to the light. The waveguide consequently shows a nonzero magnetic susceptibility at the light frequency. This way, we can move the relative permeability of semiconductor waveguides away from 1. (An optical metamaterial drawing recent attention is the stacked fishnet metamaterial [19,20]. It is, however, unsuitable for our purpose because it is difficult to combine with waveguides in such a way that it can interact with the magnetic field of TE light in the waveguide).

### 2.2. Analysis of Metamaterial Waveguides Using a Transfer Matrix Method

This section explains a transfer matrix method (TMM) for analysis of optical waveguides with metamaterials. As stated in the previous section, we take up an optical waveguide with a split-ring metamaterial (SRR array) on its surface. In following simulation, we assume guided mode as pure TE mode. However, the actual guided mode of PICs is quasi-TE. Although this does not necessarily invalidate the calculation, it is just an approximation.

To study the propagation properties of light, we divide the waveguide into multiple layers to calculate the spatial dependence of the optical field. We also replace the SRR array and its neighborhood with a (hypothetically) uniform layer with appropriate values of permittivity and permeability as shown in Figure 1b. This metamaterial uniform layer has anisotropy derived from the operation of the SRR. Therefore, we start the analysis with the permittivity and permeability tensors in an anisotropic material given by

(1) $$\tilde{\varepsilon }=\left(\begin{array}{ccc} \varepsilon_{x} & 0 & 0\\ 0 & \varepsilon_{y} & 0\\ 0 & 0 & \varepsilon_{z} \end{array}\right),\;\tilde{\mu }=\left(\begin{array}{ccc} \mu_{x} & 0 & 0\\ 0 & \mu_{y} & 0\\ 0 & 0 & \mu_{z} \end{array}\right),$$

where $\tilde{\varepsilon }$ and $\tilde{\mu }$ is the permittivity tensor and permeability tensor in each layer of the waveguide (all layers except the metamaterial uniform layer are isotropic). Using these tensors, we write the Maxwell’s equations as

(2) $$\begin{array}{c} \nabla \times H=j\omega \varepsilon_{0}\tilde{\varepsilon }E\\ \nabla \times E=-j\omega \mu_{0}\tilde{\mu }H \end{array}$$

Using Equation (2) and $\partial_{z}=j\beta$, the equations for TE-mode light can be written as

(3) $$\frac{\partial^{2}E_{x}}{\partial y^{2}}+\left({k_{0}}^{2}\varepsilon_{x}\mu_{z}-\beta^{2}\frac{\mu_{z}}{\mu_{y}}\right)E_{x}=0$$

(4) $$H_{z}=\frac{1}{j\omega \mu_{0}\mu_{z}}\frac{\partial E_{x}}{\partial y}$$

where *β* is the propagation constant in the device along *z* direction, $k_{0}=\omega \sqrt{\mu_{0}\varepsilon_{0}}=2\pi /\lambda$ is the free-space propagation constant, *E_t_* and *H_t_* (*t* = *x*, *y*, *z*) are the electric field (parallel to *t* axis) and the magnetic field (parallel to *t* axis) of the light. We find from Equations (3) and (4) that *E_x_*, *H_z_*, and *β* are given by

(5) $${\left(\begin{array}{c} E_{x}\\ H_{z} \end{array}\right)}_{top}=\prod_{m}\left(\begin{array}{cc} \cosh\left(\beta_{m}d_{m}\right) & \frac{j\omega \mu_{0}\mu_{zm}}{\beta_{m}}\sinh\left(\beta_{m}d_{m}\right)\\ \frac{\beta_{m}}{j\omega \mu_{0}\mu_{zm}}\sinh\left(\beta_{m}d_{m}\right) & \cosh\left(\beta_{m}d_{m}\right) \end{array}\right){\left(\begin{array}{c} E_{x}\\ H_{z} \end{array}\right)}_{bottom}$$

(6) $$\beta_{m}=\sqrt{\beta^{2}\left(\mu_{zm}/\mu_{ym}\right)-{k_{0}}^{2}\varepsilon_{xm}\mu_{zm}}$$

where *ε_xm_* is the *x* element of the relative permittivity tensor, *μ_ym_* and *μ_zm_* are the *y* and *z* elements of permeability tensors in the *m*-th layer, respectively, and *d_m_* is the thickness of the *m*-th layer.

At optical frequencies, the permeability tensors can be expressed using the identity matrix except in the metamaterial uniform layer. Assuming an exponential decrease in *E_x_* and *H_z_*, outside a waveguide, we can solve Equation (5) and obtain the effective refractive index (= *β/k*_0_). Combining this method with the conventional equivalent index method enables one to analyze the operation of the metamaterial waveguides. Our next task is to determine the values of *ε_x_*, *μ_y_*, and *μ_z_* of the metamaterial uniform layer.

### 2.3. Permittivity and Permeability of the Metamaterial Uniform Layer

**Figure 2:**
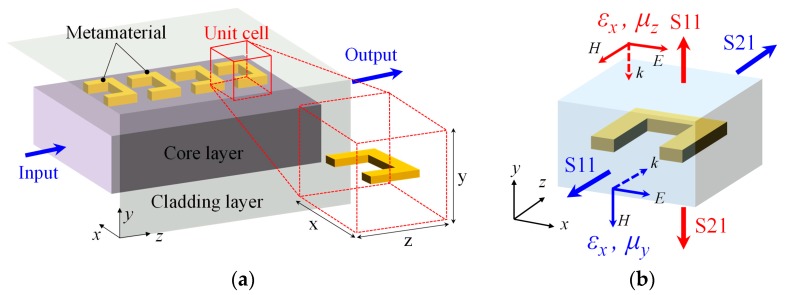
(**a**) Optical waveguide with a split-ring metamaterial on its surface. A uniform layer is substituted for the metamaterial and its neighborhood; (**b**) Retrieval of permittivity and permeability values from S parameter.

The effective values of *ε* and *μ* of the metamaterial uniform layer can be determined as follows. Consider a unit cell of the metamaterial uniform layer for each SRR of the metamaterial (Figure 2a). The unit cell is a cuboid whose length *z* and width *x* are equal to the array pitch of SRRs. Height *y* is set to an appropriate value of about one quarter or less of the wavelength, as shown later. The equivalent value of *ε* and *μ* of the unit cell (therefore the metamaterial uniform layer) can be obtained from the *S*-parameter of the SRR calculated for incident electromagnetic waves (light) introduced along *x*-, *y*-, and *z*-directions (Figure 2b) and given by [21]

(7) $$\sqrt{\varepsilon_{i}}\sqrt{\mu_{j}}=\frac{1}{kd}Arccos\left[\frac{1}{2S_{21}}\left(1-S_{11}{}^{2}+S_{21}{}^{2}\right)\right]$$

(8) $$\frac{\sqrt{\mu_{j}}}{\sqrt{\varepsilon_{i}}}=\frac{\sqrt{{\left(1+S_{11}\right)}^{2}-S_{21}{}^{2}}}{\sqrt{{\left(1-S_{11}\right)}^{2}-S_{21}{}^{2}}}$$

where *k* is the wave number of light. For example, *ε_x_* and *μ_z_* can be calculated from the set of *S*_11_ and *S*_21_ shown in red in Figure 2b. This way, the permeability and permittivity tensors of the metamaterial uniform layer can be obtained in consideration of anisotropy.

In the analysis, there is one indefinite parameter, i.e., the thickness of the metamaterial uniform layer. We adjusted this value so that the calculated transmission characteristics of the device would be consistent with the experimentally measured data.

### 2.4. Example of ε-μ Extraction from Experimental Results

What is common in optical metamaterials is that it is important but difficult to find separately the values of their permittivity *ε* and permeability *μ* (i.e., *ε*-*μ* extraction) from given experimental data of light transmission. This is because *ε* and *μ* are included in the refractive index of the material in the form of symmetric polynomial (*ε*)^1/2^(*μ*)^1/2^. Although a few studies are reported to introduce non-unity permeability to optical waveguides [22,23], they have only stayed in theory and not proceeded to actual experiments to confirm the generation of non-unity relative permeability. At the present stage, no reliable method of *ε*-*μ* extraction is reported to our knowledge. To overcome this situation, we propose a method of extracting separately the permittivity and permeability of waveguide-based optical metamaterials [24].

Now, we have four unknowns, namely the real and imaginary parts of *ε_x_* and *μ_y_*. To find them, we set up four equations by measuring two set of the amplitude and phase information of light transmission in the waveguide. The outline of the method is as follows.

**Figure 3:**
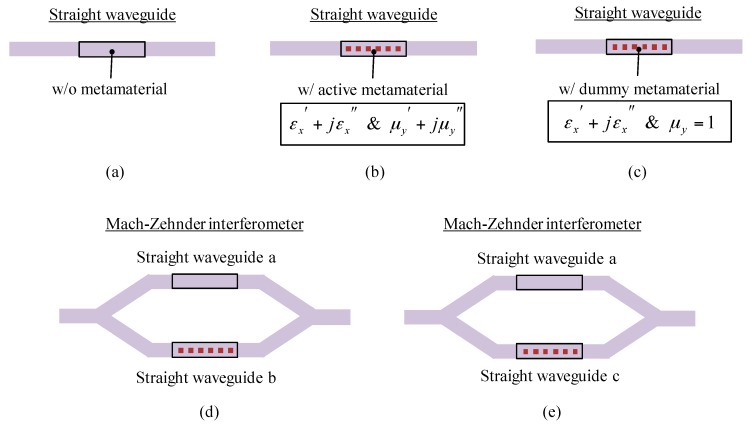
Waveguide elements needed for *ε*-*μ* extraction. (**a**) Straight waveguide without TGM; (**b**) straight waveguide with active TGM; (**c**) straight waveguide with dummy TGM, (**d**) MZI consisting of waveguides a and b connected with Y branches; and (**e**) MZI consisting of waveguides a and c.

We first prepare two metamaterials, namely (1) active metamaterial: a metamaterial to be evaluated, consisting of SRRs that interact magnetically with input light; and (2) dummy metamaterial: a metamaterial consisting of SRRs that has almost the same as active metamaterial’s but has no interaction with input light. We then prepare three waveguides (a) without metamaterial; (b) with active metamaterial on its surface); and (c) with dummy metamaterial on its surface (see Figure 3).

The active metamaterial has an effect on both *ε_x_* and *μ_y_* around the frequency of light because of magnetic interaction with the light. In contrast, the dummy metamaterial has an effect only on *ε_x_* and does not move *μ_y_* from 1. Using these waveguides, we prepare two Mach–Zehnder interferometers (MZIs) consisting of (d) waveguides a and b and (e) waveguides a and c that are connected with Y branches (see Figure 3). The transmission of light passing through the MZIs depends on a phase difference between two waveguides. We can find *ε_x_* and *μ_y_* by measuring the transmission intensity of light through waveguides a, b, and c and MZIs d and e; that is,

- Complex permittivity *ε_x_* can be calculated from the transmission data of waveguide a, waveguide c, and MZI e, where *μ_y_* is set to 1, and
- Complex permeability *μ_y_* can be calculated from the transmission data of waveguide a, waveguide b, and MZI d, using the value of *ε_x_* obtained in I.

**Figure 4:**
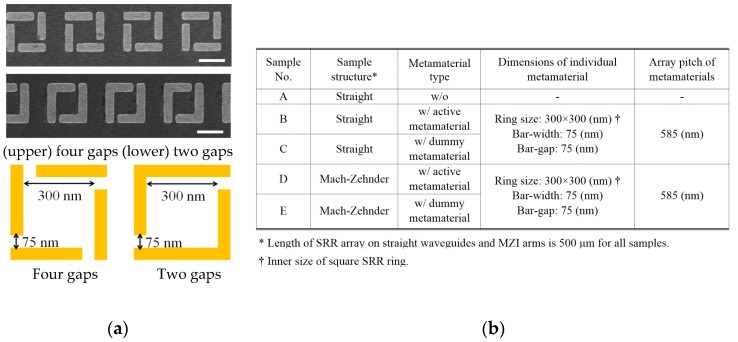
(**a**) Oblique view of four-gap and two-gap SRRs used for *ε*-*μ* extraction. Scanning electron microscopy (SEM) photograph and schematic view with dimensions; (**b**) All parameters used in the experiment.

We show an actual example of *ε*-*μ* extraction using our method. As the active metamaterials, we used an array of four-gap SRRs (see Figure 4a) that were designed to resonate at about 193 THz and interact with 1.5-μm light travelling in the waveguide. The dummy metamaterial was composed of two-gap SRRs whose shape and dimensions were almost the same as those of the four-gap SRR except for the number of gaps (also see Figure 4a). The two-gap SRR had a far higher resonant frequency and no interaction with 1.5-μm light. The array pitch was set to 585 nm. Thus, we prepared three waveguides with and without SRRs and two MZIs with active and dummy waveguide arms.

**Figure 5:**
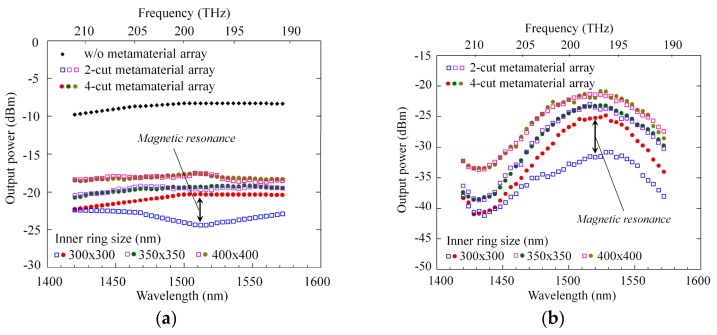

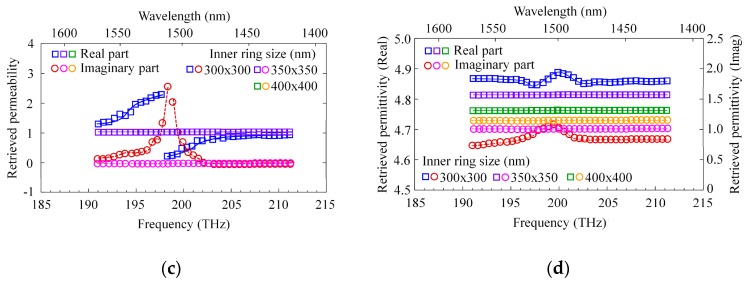
(**a**) Transmission spectra measured for (**a**) three waveguides and (**b**) two Mach–Zehnder interferometers (MZIs) with different ring size; (**c**,**d**) Retrieved relative permeability (real and imaginary parts) of substituting layer, plotted as a function of light frequency.

Figure 5a,b show the transmission intensity measured as a function of light wavelength for the three waveguides and two MZIs. Using these results, we calculated separately the permeability *μ_y_* and permittivity *ε_x_* of the substituting layer. In calculation, the thickness of the substituting layer was set to 230 nm. Figure 5c,d depicts the value of permeability *μ_y_* as a function of light frequency. The real part of *μ_y_* showed a variation from 0.1 to 2.2 on both side of 198 GHz (1510-nm wavelength) for a 300 × 300-nm-SRR substituting layer. Thus, we were able to confirm that a SRR array moved the permeability of its adjacent semiconductor region away from 1. Our method of *ε*-*μ* extraction is very useful in developing metaphotonic devices that can control the permeability as well as the permittivity of optical semiconductor materials.

## 3. Specific Metamaterial to Control Permeability of Semiconductor Photonic Devices

### 3.1. Controlling Permeability Using Carrier Accumulation of Semiconductors

We next devise a method to control the magnetic property of the SRR with an external electric signal. This is required to vary the permeability of waveguides with an electric signal. The magnetic property of the SRR depends on the resonant frequency of the SRR, and therefore our task is to make a controllable SRR whose LC parameters can be changed with an electric signal. A few methods are considered to create controllable SRRs with variable magnetic property. The most effective method is to directly vary the shape of the split ring, thereby varying its LC parameters, using micro-electro mechanical system (MEMS) actuators [25]. However, it cannot work fast because of its mechanical operation. Another method is to combine SRRs with a semiconductor material, such as amorphous silicon and vanadium oxide, and control the property of the material with an external signal to change SRR operation [26,27,28,29,30]. It is, however, not easy to achieve desired material properties with good controllability. Moreover, these methods are incompatible with GaInAsP/InP-based photonic devices.

**Figure 6:**
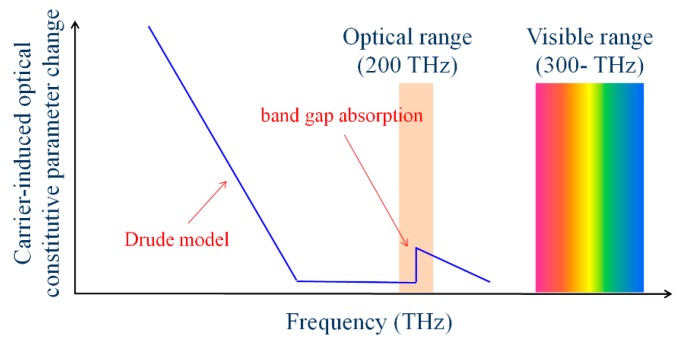
Rate of variation in optical properties, outlined as a function of light frequency.

As a new method, we consider controlling the magnetic property of metamaterials by varying the SRR gap capacitance, making use of carrier accumulation of semiconductors. A semiconductor varies its optical properties (permittivity and conductivity) as a function of its carrier density. We make use of this variation to control the gap capacitance of SRRs. In semiconductors, the rate of the variation depends on the frequency of light as outlined in Figure 6. It can be expressed with a Drude model at low frequencies where the frequency of light (or electromagnetic wave) is far smaller than the reciprocal of the relaxation time of carriers. In this frequency region, the optical properties can be largely controlled by modulating carrier density, and this can be used to make electrically controllable metamaterials for microwave applications [31,32,33]. As frequency increases, the optical properties become independent of carrier density. However, the variation occurs again in the neighborhood of the frequency that corresponds to the bandgap energy of the semiconductor. This is due to a bandgap shift caused by the band filling, free-carrier plasma dispersion, and bandgap shrinkage effects of the semiconductor [34,35].

We calculated the variation in the refractive index and absorption coefficient of Ga_0.47_In_0.53_As as functions of the wavelength of light, with carrier density as a parameter. In this simulation, three effects, i.e., the band filling, bandgap shrinkage, and free-carrier absorption, were assumed to make substantial contributions to the total changes in the refractive index and absorption loss. In the case of band filling, there is a finite probability that a state in the conduction band is occupied by an electron and/or a state in the valence band is empty. Thus, the band-filling-induced change in absorption is given by

(9) $$\begin{array}{ll} \Delta \alpha \left(N,E\right)= & \frac{C_{hh}}{E}\sqrt{E-E_{g}-\Delta E_{g}\left(N\right)}\left[f_{v}\left(E_{ah}\right)-f_{c}\left(E_{bh}\right)\right]+\frac{C_{lh}}{E}\sqrt{E-E_{g}-\Delta E_{g}\left(N\right)}\left[f_{v}\left(E_{al}\right)-f_{c}\left(E_{bl}\right)\right]\\ & -\frac{C_{hh}}{E}\sqrt{E-E_{g}}-\frac{C_{lh}}{E}\sqrt{E-E_{g}}+\Delta \alpha_{free}\left(N,E\right) \end{array}$$

where *E* = *hω*/2*π* is the photon energy; *N* is the concentration of free electrons; *E_g_* is the bandgap energy; *C_hh_* and *C_lh_* are constants involving materials parameters, matrix elements between periodic parts of the Bloch states at the band edges, and fundamental constants for heavy and light holes; *f_c_*(*E_b_*) is the probability of a conduction band state of energy *E_b_* being occupied by an electron; and *f_v_*(*E_a_*) is the probability of a valence band state of energy *E_a_* being occupied by an electron.

For a given photon energy, the values of *E_a_* and *E_b_* are uniquely defined. Here we also introduce the bandgap shrinkage and free-carrier absorption, which cause a rigid translation of the absorption curve. The bandgap shrinkage Δ*E_g_*(*N*) is given by [36]

(10) $$\Delta E_{g}\left(N\right)=-\left(\frac{e}{2\pi \varepsilon_{0}\varepsilon_{s}}\right){\left(\frac{3}{\pi }\right)}^{\frac{1}{3}}N^{\frac{1}{3}}$$

where *ε*_0_ is the permittivity of free space and *ε_s_* is the relative static dielectric constant of the semiconductor. On the other hand, an expression for the free-carrier absorption Δ*α*_free_(*N,E*) is

(11) $$\Delta \alpha_{\mathrm{free}}\left(N,E\right)=4.25\times 10^{-20}e^{-\frac{3.66E}{1.6\times 10^{-19}}}\times N$$

The real and imaginary parts of the refractive index are related by the Kramers–Kronig integrals. Therefore, the change in refractive index Δ*n* was finally calculated by applying the following integral to Δ*α*:

(12) $$\Delta n\left(N,E\right)=\frac{2c\hbar }{e^{2}}P\int_{0}^{\infty }\frac{\Delta \alpha \left(N,X\right)}{X^{2}-E^{2}}dX$$

where *P* indicates the principal value of the integral.

**Figure 7:**
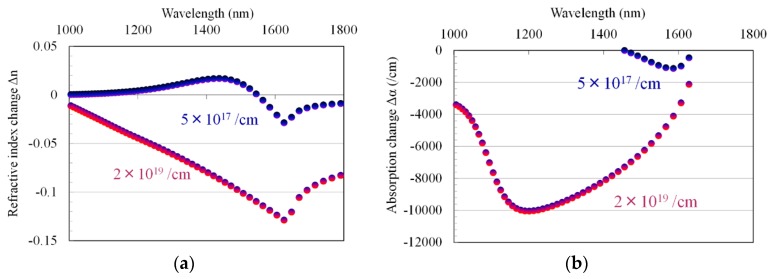
Variation in refractive index (**a**) and absorption coefficient (**b**) of Ga_0.47_In_0.53_As as functions of wavelength of light, with carrier density as a parameter.

Figure 7a,b shows the calculated refractive index and absorption coefficient of Ga_0.47_In_0.53_As as a function of the wavelength of light, respectively; carrier concentration is the parameter. A large variation is observed at 1500–1600 nm corresponding to the bandgap energy of Ga_0.47_In_0.53_As. We use this variation to control permeability values of metamaterials. There are two main factors that have great effect on resonance frequency of the SRR in our device. One is dissipation due to the substrate free carrier absorption within the split gap, and the other, which is a dominant factor in our device, is a fringing capacitance within the split gap. The capacitance is related to the dielectric function of the semiconductor substrate due to the field lines fringing into the material. When the carrier concentration of the semiconductor substrate varies, the capacitance change results in a resonance shift. As stated above, the real part of GaInAs’s dielectric function decreases as the carrier concentration increases. A decrease of the real component of the substrate’s dielectric function will decrease the capacitance within the split gap, and therefore shift the resonance to a higher frequency (or shorter wavelength). This is a principle for the resonance-frequency shift in this study.

### 3.2. Specific Metamaterial Suructure for Electrical Control of Permeability

**Figure 8:**
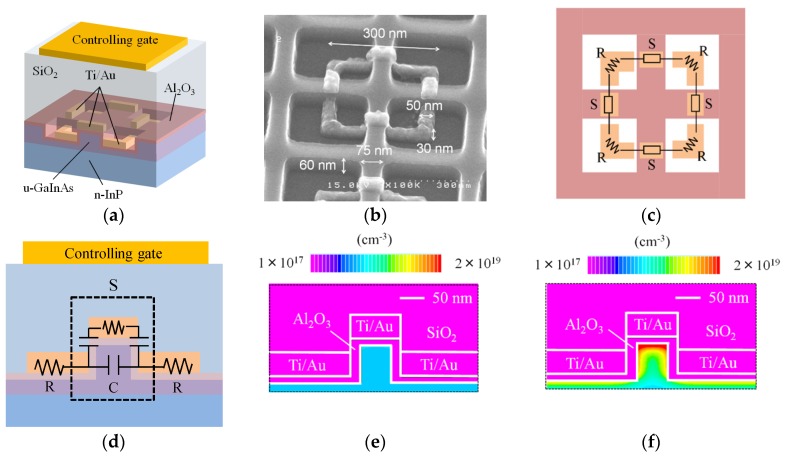
Gate-controlled metamaterial. (**a**) Three dimensional view of individual metamaterial; (**b**) SEM photograph around metal ring; (**c**) schematic top view of ring circuit; (**d**) equivalent circuit of gap capacitance; and (**e**) electron density induced in GaInAs fin at gate voltage = 0 V and (**f**) 20 V, simulated with three-dimensional TCAD.

On the basis of this idea, we designed a gate-controlled metamaterial that is compatible with GaInAsP/InP-based photonic devices (see Figure 8a,b). The details are as follows:

- A GaInAs thin layer is formed on the GaInAsP/InP-based optical waveguide and etched into grid-shaped fins;
- An Al_2_O_3_ layer covers the surface of the GaInAs fins;
- To make the SRR, a metal (Ti/Au) ring is formed on the surface in a manner such that it wraps the Al_2_O_3_-covered fins and is cut at the edges of the fin. The gap capacitance of the SRR exists at the places where the metal ring is cut (Figure 8c,d). The metal ring and fin form a structure similar to that of a triple-gated, three-dimensional transistor [37,38];
- A controlling gate is placed above the SRR (not illustrated). It is coupled capacitively with the SRR.

Now, let us apply a positive voltage to the controlling gate. Then, the SRR acts as a floating triple-gate electrode in addition to its role as an LC resonator. Applying the controlling voltage raises the dc potential of the SRR, thereby inducing electrons in the GaInAs fin, and thereby varies the gap capacitance of the SRR. Thus, we can control the resonance, and therefore the magnetic response, of the SRR. By arraying the SRRs on a semiconductor waveguide, we can control the permeability of the waveguide with the gate voltage. We call an array of the SRRs a ‘tri-gate metamaterial (TGM)’ because of the triple gate structure of the SRR.

We simulated the distribution of induced electron density in the fin under a given gate bias with the aid of a 3-dimensional TCAD device simulator. Here, we took into consideration the Poisson equation, electron and hole continuity equation, parallel electric field-dependent mobility model, concentration-dependent carrier mobility model, Shockley–Read–Hall (SRH) recombination model, material-dependent band parameter model, and Fermi–Dirac statistics model. Figure 8e,f shows the results, with red-to-blue color gradation, calculated for controlling gate voltages V_g_ = 0 V and V_g_ = 20 V. The electron density in the fin is effectively modulated by the gate voltage because of the triple-gate structure. The intrinsic speed of this device is limited by electron-accumulation/extinction speed in the fin in the on-off driving of the gate voltage. From time-dependent TCAD simulation results, electron accumulation and extinction speeds were estimated to be 1.5 and 2.0 ps, respectively [39]. This result indicates that the operation speed over 50 GHz can be achieved with this modulator (no traveling-wave electrode will be necessary because a MZ arm length of the device will ultimately become less than 50 μm).

**Figure 9:**
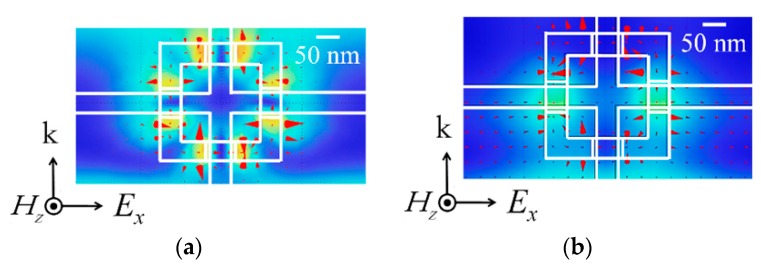
Simulated magnetic field distribution around an individual tri-gate metamaterial (TGM) having appropriate dimensions at (**a**) 190 THz and (**b**) 230 THz. The distribution of vector field is also visualized by a red arrow.

The SRR we used has four gaps in series and therefore a small gap capacitance. This enables the SRR to resonate at the high frequency of 1.55-μm light. The resonant frequency of the SRR can be controlled with the gate voltage, but its variable range is not wide because the gap capacitance can be varied only a little with gate-induced electrons in the fin. Therefore, it is important to design the SRR such that its resonant frequency is in the neighborhood of the frequency of 1.55-μm light. To determine the appropriate dimensions of the SRR, we simulated interaction between TGM and TE light for various dimensions of the SRR. In the simulation, (i) the finite element method was used; (ii) the conductivity of the metal ring was defined according to the Drude model. Figure 9a,b depicts the distributions of magnetic fields around an individual TGM having appropriate dimensions at 190 THz and 230 THz, respectively. At 190 THz, the LC resonance is produced by magnetic interaction between the TGM and light. In this resonance, a loop current, therefore a magnetic dipole moment, is induced in each SRR of the TGM, and this modulates the permeability of the waveguide arm. At 230 THz, the Mie resonance is produced by plasma oscillation in the metal ring and has no effect on the permeability. The distribution of vector fields is also visualized by a red arrow. From the results of simulation, we determined the dimensions of the SRR for experimental devices. The dimensions we used were: (i) ring-wire width = 50 nm; (ii) fin width including Al_2_O_3_ layer = 75 nm; and (iii) fin height = 60 nm, (iv) outer size of ring = 300 × 300 nm (also see Figure 8b).

## 4. Permeability-Controlled Optical Modulator for Integrated Optics

### 4.1. Fabrication and Concept of Device

**Figure 10:**
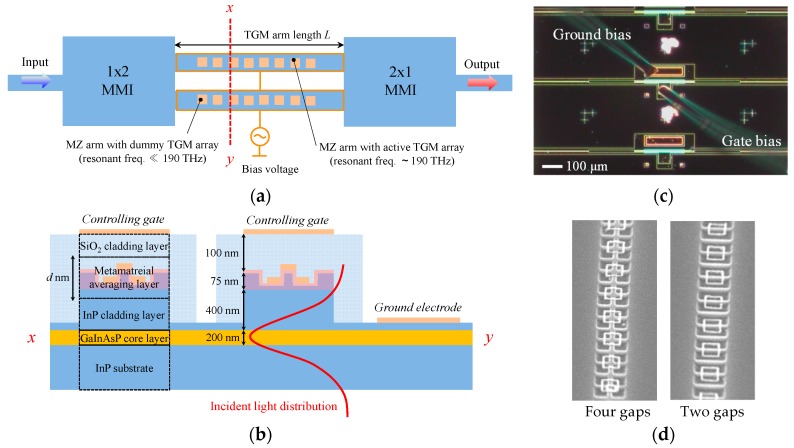
Structure of permeability-controlled optical modulator consisting of waveguide-based GaInAsP/InP Mach–Zehnder interferometer with TGM attached on waveguide arm. (**a**) Plan view of modulator; and (**b**) cross section of waveguide arm; (**c**) Fabricated permeability-controlled optical modulator observed with optical microscope; (**d**) Enlarged active TGM and dummy TGM observed with scanning electron microscope.

Using TGM stated in Section 3.2, we made a permeability-controlled optical modulator that can be used at a wavelength of 1.55 μm. Figure 10a,b shows the structure. The modulator consists of a waveguide-based GaInAsP/InP Mach–Zehnder interferometer (MZI) with TGM attached on the waveguide arm [39]. The TGM consists of the gate-controlled SRRs arranged in a row on the arm. Applying a controlling gate voltage varies the permeability, and therefore the refractive index, of the arm, and this produces a phase difference between two optical paths in the arms. Thus, we can modulate the amplitude of output light with the gate voltage. The operation is almost the same as that of existing interferometric modulators. The difference is that our device varies the permeability, not the permittivity, of the waveguide arm to alter the phase of light waves.

The process of device fabrication was as follows. The starting material was a (100) oriented n-type InP substrate. On the substrate, three layers, namely, an undoped Ga_0.23_In_0.77_As_0.5_P_0.5_ core layer (bandgap wavelength = 1.22 μm and thickness = 200 nm), n-type InP clad layer (400-nm thick and 5 × 10^17^/cm^3^), and undoped Ga_0.47_In_0.53_As surface layer (75-nm thick) were formed in this order using metal-organic-chemical-vapor deposition. (These layers were selectively etched in the final step to form the pattern of MZI waveguides). The TGM was made on the place where the MZI arm was to be formed. To make the TGM, a grid of GaInAs fins was first formed using electron-beam lithography and CH_4_/H_2_ reactive ion etching, and then a 10-nm-thick Al_2_O_3_ layer was deposited using magnetron sputtering. Next, 300 × 300-nm square rings (5-nm-thick Ti and 20-nm-thick Au) are made using lithography, electron-beam deposition, and a lift-off process. The triple-gate structure was automatically formed at the crossing points between the SRR rings and fins (see Figure 8b). After that, a 100-nm-thick SiO_2_ layer was formed to cover the surface, using plasma-enhanced chemical vapor deposition. Then, metal electrodes (10-nm-thick Ti and 200-nm-thick Au) were deposited on the top of the device and on the InP cladding layer. The top electrode is the controlling gate. The gate voltage is applied between the two electrodes. The final step is the formation of the MZI pattern. A 100-nm SiO_2_ layer was formed on the surface with plasma-chemical-vapor deposition and then formed into the MZI pattern with electron-beam lithography and reactive-ion etching of the SiO_2_. After that, the GaInAs/InP/GaInAsP triple layer was selectively etched to make the MZI waveguide structure, using the SiO_2_ pattern as a mask for reactive-ion etching.

Figure 10c shows the plan view of the fabricated optical modulator observed with an optical microscope. A TGM is formed on one arm of the MZI by arraying the gate-controlled SRRs in a row on the arm. We hereafter call this arm an ‘active TGM arm’. The length of the TGM arm (i.e., the length of the SRR array along the arm) was set to 200 μm. To maintain the balance between the two arms, we formed a dummy TGM of 200-μm length on the other arm of the MZI. The dummy TGM has almost the same structure as that of the active TGM arm except that its SRR has two gaps instead of four gaps (see Figure 10d). The dummy TGM has no magnetic interaction with 1.55-μm light because its resonance frequency is far lower than that of 1.55-μm light.

In our MZI device, a phase difference Δφ between two arms for a given controlling gate voltage is given by

(13) $$\Delta \varphi =\frac{2\pi }{\lambda }\left(n_{0}^{active}+\Delta n_{1}^{active}+\Delta n_{2}^{active}-n_{0}^{dummy}-\Delta n_{2}^{dummy}\right)L=\frac{2\pi }{\lambda }\left(n_{0}^{active}-n_{0}^{dummy}+\Delta n_{1}^{active}\right)L$$

where *λ* is the wavelength of light, *L* is the length of the TGM arms, $n_{0}^{active(dummy)}$ is the effective refractive index of the active (and dummy) TGM arm at gate voltage = 0, $\Delta n_{1}^{active}$ is the variation in the refractive index of the active TGM arm that is induced by the TGM’s resonance shift due to a positive gate voltage, and $\Delta n_{2}^{active(dummy)}$ is the variation in the refractive index of the active (and dummy) TGM arm due to parasitic factors such as variations in effective permittivity and free-carrier absorption induced by a positive gate voltage. The term of $\Delta n_{2}^{active}-\Delta n_{2}^{dummy}$ in Equation (14) can be ignored because both active and dummy TGM arms have almost the same structure. The phase difference Δφ is therefore determined by $n_{0}^{active}-n_{0}^{dummy}$ and $\Delta n_{1}^{active}$, where $n_{0}^{active}-n_{0}^{dummy}$ is produced by the difference of permeability between the active and dummy TGMs at gate voltage = 0, and $\Delta n_{1}^{active}$ is produced by a variation in permeability of the active TGM induced by a positive gate voltage. Thus, the transmission characteristics of light in the MZI are all related to the permeability variation in the active TGM.

### 4.2. Operation of Permeability-Controlled Optical Modulator

We measured the transmission of light in our modulators. In measurements, TE-polarized light was sent from a tuneable laser to the device through a polarization controller. The wavelength was 1.55 μm. The output light from the other end of the device was gathered by a lens to observe the near-field pattern. After confirming that the output light was in a single mode, we measured its intensity as a function of gate voltage for the SRRs.

**Figure 11:**
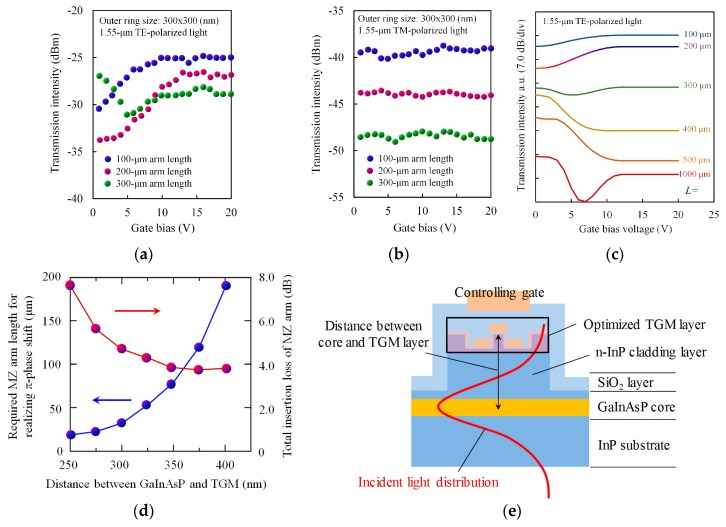
Transmission intensity of permeability-controlled modulator as a function of gate voltage; (**a**) Measured results for TE light; (**b**) measured for TM light; and (**c**) simulated transmission characteristics with TGM arm length as a parameter; (**d**) Simulated performance limit of permeability-controlled modulator; required MZ arm length for realizing π-phase shift together with a total insertion loss of the MZ arm as a function of the distance between the GaInAsP core and optimized TGM layer; (**e**) Schematic image of cross sectional image of device.

Figure 11a shows the TE-mode transmission characteristics of the device as a function of controlling gate voltage. Here we had prepared the three devices with different TGM arm length of 100 μm, 200 μm, and 300 μm. The transmission intensity changed as the gate voltage increased, and an extinction ratio of 6.9 dB was obtained with a gate voltage swing of 2.0-to-12.0 V for the device with arm length of 200 μm. This predicted that the designed TGM would successfully interact with 1.55-μm TE-mode light. We also measured the device for TM-polarized input light. The transmission intensity was almost constant and independent of gate voltage in every device (Figure 11b). This is because TM light has no magnetic interaction with TGM because its magnetic field is parallel to the SRR ring plane, and therefore no variation is produced in permeability. In general, integrated photonic devices are used in TE-mode operation because light from laser diodes is TE-polarized. Therefore, the insensitivity to TM waves would not be a problem in practical application.

In parallel with experiments, we calculated the transmission characteristics of the device in the following procedure:

- Calculate gate-induced electron distribution in the fin as a function of gate voltage, using a three-dimensional semiconductor device simulator (Silvaco Device3D, Santa Clara, CA, USA);
- Calculate the S parameter of one unit cell of the TGM by analysing light propagation over the unit cell with the aid of an electromagnetic simulator (Comsol Multiphysics, Comsol, Burlington, MA, USA);
- The TGM and its neighborhood (its adjacent part of the waveguide) can approximately be considered as a single uniform layer. Under this approximation, calculate the effective permeability and permittivity of the layer from the S parameter obtained in (ii), using the method described in Section 2.3;
- Conduct waveguide analysis to evaluate the transmission characteristics of the permeability-controlled MZ modulator, replacing the TGM and its neighborhood with the single layer. The waveguide analysis can be easily performed using metamaterial waveguide theory described in Section 2.2.

Figure 11c shows the simulated transmission intensity of the device having the same structure as that of the experimental one, calculated as a function of gate voltage (0–20 V). The length of the TGM arm was changed (100–1000 μm) as a parameter. At a zero gate voltage, the active TGM arm interacts magnetically with light and varies its relative permeability from 1, whereas the dummy TGM arm does not interact and maintains its relative permeability of 1. Consequently, a phase difference occurs between the two arms. Increasing the controlling gate voltage moves the active TGM away from the resonance, and therefore the relative permeability of its arm approaches 1, resulting in a decrease in the phase difference. As a result, the transmission intensity changes with gate voltage. The simulated and experimental data are consistent with each other. Simulation predicts that a maximum extinction ratio of 15 dB can be expected for a 500-μm device, which is a performance obtained by controlling permeability only. The modulation in the device saturates at 15–20 V gate voltage. This is because the carriers induced in the fin by the gate voltage saturates at a density of about 10^−19^ cm^−3^, corresponding to a gate voltage of 15–20 V.

Using a modified device structure will enable a higher device performance. As a future target, we calculated the performance limit of the permeability-controlled modulator when using the maximum permeability change. Figure 11d summarizes the results, i.e., a required MZ arm length for realizing π-phase shift together with a total insertion loss of the MZ arm as a function of the distance between the GaInAsP core and optimized TGM layer. In our modulator, light traveling along the GaInAsP/InP waveguide extends through the *n*-InP cladding layer into the TGM layer and interacts to ensure magnetic response (see Figure 11e). Therefore, the thickness of the *n*-InP cladding layer greatly affects the performance of the modulator. As shown in Figure 11d, extremely short device length (<50 μm) can be realized because changing permeability in addition to permittivity produces large modulation of the refractive index of semiconductors. If we set the interaction distance to be 300 nm, the π-phase shift can be obtained with the MZ arm length of 35 μm with the total insertion loss of about 4.5 dB. This will transform conventional optical modulators (e.g., electro-absorption modulator) into small-sized, high-performance devices for photonic integration.

## 5. Summary

There are two techniques at present to adjust the optical properties of semiconductors to the needs of device designers. One is ‘bandgap engineering’, that is, varying the bandgap appropriately in a device (e.g., heterostructure and quantum wells in lasers and photodetectors), and the other is ‘permittivity engineering’, or varying the permittivity appropriately in a device (e.g., core/clad structure in waveguides). Both can easily be achieved by adjusting the composition of semiconductors, that is, by using mixed crystals. Now, as the third technique, let us consider ‘permeability engineering’. Varying the optical permeability of semiconductors is not easy and cannot be achieved with mixed crystals. To show the feasibility of permeability engineering, we have shown by experiments that a SRR metamaterial on a GaInAsP waveguide moves the effective relative permeability of its adjacent part of the waveguide away from 1 at optical frequencies. To control the permeability of waveguides, we have made a SRR whose magnetic property can be varied with gate voltage. As an application of the variable-permeability waveguide, we have demonstrated an optical modulator consisting of a GaInAsP/InP Mach–Zehnder interferometer for use at an optical communication wavelength of 1.55 μm. This device changes the permeability, not permittivity, of its waveguide arm with the gate voltage, thereby changing the refractive index of the arm to modulate the intensity of output light. In addition, for the study of permittivity engineering, we have developed a method of extracting separately the values of effective permeability and permittivity of SRR-attached waveguides from the experimental data of light transmission.

The optical modulator we made is an application of permeability control in the first quadrant of the *ε*-*μ* plane (*ε* > 0, *μ* > 0). We can consider applications in other quadrants, especially the third quadrant (*ε* < 0, *μ* < 0) where the refractive index is negative. If the *ε*-*μ* values of a waveguide prism can be switched between the the first quadrant (normal prism region) and the third one (superprism region), then wide-angle optical deflection will be achieved. The waveguide superprism has other potential applications such as highly efficient wavelength demultiplexing.

Controlling permeability as well as permittivity will develop conventional optical devices into small-sized, high-performance devices and produce novel photonic devices with sophisticated functions for metaphotonic integration. The method of permeability control we proposed can be applied to silicon photonic devices as well as InP-based ones. We hope that our method will contribute to the development of next-generation photonic integrated circuits and application systems.
